# Colonization of minds: Ukraine between Russian colonialism and Western Orientalism

**DOI:** 10.3389/fsoc.2023.1206320

**Published:** 2023-10-30

**Authors:** Galyna Kotliuk

**Affiliations:** Heinrich Böll Foundation, Kyiv, Ukraine

**Keywords:** gender, Ukraine, Russian war against Ukraine, colonialism, Orientalism

## Abstract

This article focuses on investigating how Russian colonialism has re-invented Ukrainian identity and framed its perception both in Ukraine and in Western societies. Although postcolonial approaches to studying power hierarchies have contributed a lot to our understanding of global inequalities, Ukraine has long been excluded from this interdisciplinary, complex discussion. Such exclusion has created a knowledge gap, which this study aims to address by analyzing the evolution of gender identities and roles in Ukrainian society and critical rethinking of the existing body of knowledge. This article demonstrates the degree to which European societies perceived Ukrainians as Orientalized passive objects, which went in line with the Russian imperialistic ideology of *russkii mir*, examines its influence on gender identities in Ukraine and, finally, proves that the strengthening of Ukrainian civil society as well as women’s and LGBTQ+ rights is a part of the decolonial processes that were instigated by the Russian war against Ukraine.

## Introduction

Ever since Postcolonial Studies was established as a cross-disciplinary approach to analyzing political, military, economic, and cultural dimensions of colonial practices and politics, the field has produced a thorough study of the interaction between the colonial powers and their former colonies as well as a meticulous investigation of intricate relations between the colonizer and the colonized. What is surprising, however, is that, despite covering in their research an extensive geography of the colonized territories, which include the Americas, Africa, Australia, the Middle East, and Asia, postcolonial discourses are leaving a significant blank space in Europe, thus neglecting to address the territories that used to be known as the “Second World” during Soviet time. Such a paradigm completely excludes Russian colonialism from the scope of its research, thus failing to critically approach and study it. For example, Schwarz and Ray’s highly popular *A Companion to Postcolonial Studies* (2005) does not recognize Russia or the Soviet Union as a colonial superpower and mentions “Soviet imperialism” (Pease, 2005: 218) only as an authorized justification of American “neo-colonialism” (Pease, 2005: 218) during the time of the Cold War. *The Postcolonial Studies Reader*, edited by Ashcroft et al. (2003) [who are also the authors of another highly influential study, *The Empire Writes Back: Theory and Practice in Postcolonial Literatures* (1989)], is also very reluctant to talk about Soviet imperialism, only barely mentioning Soviet soft power as a means of promoting the “Soviet brand” (456; 488) without even attempting to analytically study Soviet colonialism and the role Russian imperialism played in it. McLeod’s *The Routledge Companion to Postcolonial Studies* (2007) focuses exclusively on European colonial powers (British, French, Spanish, and Portuguese), thus completely ignoring the imperial nature of the Russian Empire and its successor, Soviet Russia. In Western academia, this silence was partially broken by Ewa Thompson’s *Imperial Knowledge* (2000) and has been occasionally addressed by Ukrainian scholars (Velychko, 2002; Riabczuk, 2013; Ageyeva, 2021; Yermolenko, 2021). However, Russian colonialism seems to be excluded from the postcolonial discussion in Western academic discourse, thus creating the fallacious impression that Russian colonialism as such has never existed.

This consistent (in)attentional blindness has turned the former countries of the Soviet Bloc into a mysterious gray zone—the non-existent Second World, the “non-existence” of which has been functioning as a valid excuse to ignore the biggest colonial power in the Eurasian continent. This ignorance has not only excluded the former Soviet republics from postcolonial knowledge production and exchange but also sanctioned Russian neo-imperial ambitions after the collapse of the USSR. This ambition was manifested in the illegal occupation of Moldova’s territory (1992), the First and Second Russo-Chechen Wars (1994–1996; 1999–2009), the brutal invasion of Georgia and the occupation of 20% of its territories (2008), Russia’s violent military intervention in Syria (2015), the occupation of Ukraine’s Donetsk and Luhansk oblasts, and the annexation of Crimea (2014), both of which bore little to no consequences for the Russian Federation and thus encouraged the full-scale war waged on Ukraine on 24 February, 2022. In my study, I aim to analyze the reasons the West has long been ignoring the imperial nature of the Soviet Union, which was successfully covering its own imperialism under a declared “voluntary” union of the Soviet republics, as well as the neo-imperial character of the Russian Federation, embodied in the linguocentric ideology of Russian supremacy based on the assumption of Russian exceptionalism—the *russkii mir* (Russian world). This article demonstrates the distorted image of Ukraine and Ukrainian women created at the intersection of Russian colonialism and Western Orientalism—the image according to which weak and orientalized Ukraine would fall within 96 h if the full-scale invasion took place. In this study, I further critically analyze and dismantle this constructed narrative, thus offering a new approach to understanding Ukrainian gender identities through the postcolonial lens.

### (Western) Academia and imperialism

In two of the seminal (and, indeed, most influential) works in the field of Postcolonial Studies, *Orientalism* (1978) and *Culture and Imperialism* (1993), Edward Said, while analyzing the rule of the greatest colonial powers on the European continent, completely disregards the Russian/Soviet imperial project. In *Culture and Imperialism*, Said scarcely mentions Russia as a colonial power and immediately finds an excuse to legitimize Russian colonialism, framing it as a “good version” of colonialism in comparison to one of the Western empires: “Russia, however,” as claimed by Said, “acquired its imperial territories almost exclusively by adjacence.” Unlike Britain or France, which jumped thousands of miles beyond their own borders to other continents, Russia moved to swallow whatever land or people stood next to its borders, which in the process kept moving further and further east and south.” (Said, 1994: 10). Commenting on this quote from Said’s work, I want to make three important points. First, claiming that the borders of the Russian empire “kept moving further and further east and south,” Said overlooks a significant historical reality: the Russian empire has occupied and then colonized the lands west of its borders, i.e., the territories of modern Ukraine, Belarus, Poland, and the Baltic states. Consequently, this discourse plays along with the colonial narrative that tacitly positions Ukraine within the uncontestable geographical sphere of Russia and reinforces the artificially created idea that Ukrainians inhabiting this territory are not different from native Russians. This conspicuous denial of Ukrainians’ nationhood—or, at least, a failure to recognize it as such—perpetuates the imperialistic myth of three “brother nations”—Russians, Ukrainians, and Belarusians (or Great Russians, Little Russians, and White Russians, as it is often defined within the line of Russian colonial naming vocabulary), who together comprise a “Slavic Trinity,” the all-Russian nation united under the rule of all-encompassing Russian colonial ideology and politics—the *russkii mir*. Second, talking about Russian colonialism, Said follows a widespread thought in the Western colonial discourse that a colony is a far-distant remote place that lies overseas—as in the case of India controlled by the British or French colonies in Africa—thus seemingly justifying the Russian colonialism by “adjacence” as the one juxtaposed to British and French colonizing politics that is so much criticized in his works. Third, engaging with Gramsci’s concept of *hegemony* in his *Orientalism*, Said defines “the idea of European identity as a superior one in comparison with all the non-European peoples and cultures” (Said, 2019: 7). Ironically enough, the same idea of European hegemony and superiority so much condemned by Said failed him to recognize not only Russian colonialism as such but also the fact that West European colonial dominance has always been contested by another colonial power situated east of the European borders. This prioritization of the West was likely influenced by “Russia’s purported Eurasia character” (Moore, 2001: 119), the idea that has framed Russia as “neither European nor “Asiatic” but as somehow in between, and particularly as more primitive than (Western) Europe” (Moore, 2001: 119), and thus unable to fit into the paradigm of (West) European hegemonic colonial models and presumably not capable of colonizing other nations and keeping them under its colonial rule.

Another reason, which lies at the basis of Russian immunity to any allegations of exercising their colonial aspirations, is the inherent character of Marxism in the field of Postcolonial Studies, and many researchers and scholars^1^ who engaged with postcolonial thinking regarded imperialism exclusively in Lenin’s terms as “the highest stage of capitalism^2^.” This claim of Lenin defined colonialism as an integral part of capitalism, thus exempting the USSR from the (post)colonial discussions and allowing Russia to avoid any criticism of its neo-imperial politics in the future. Ukrainian scholar and historian Stephen Velychko, studying the nature of Western ignorance of Soviet/Russian colonial expansion, makes an important observation:

“[…] influential organizations such as the League Against Imperialism (1929), The Congress of Peoples Against Imperialism (1948), the Movement for Colonial Freedom (1949), the American Committee on Africa (1953), either ignored or justified Moscow’s exercise of hegemonic power in Eurasia, imagined colonial exploitation a thing of the past in that part of the world, and focused their attentions on American and European imperialism. When the Ceylonese President at the 1955 Bandung Conference proposed that there was a Soviet colonialism that had be abolished just like any other, Nehru interjected that the subject was not on the conference agenda and closed the issue. World opinion did not consider the 1960 UN “Declaration on the Granting of independence to Colonial Countries and Peoples” applicable to the USSR.” (Velychko, 2002: 335).

The allegedly anti-capitalist character of the Soviet Union prevented Western intellectuals from seeing similarities between the capitalist and communist models and recognizing the fact that “the destructive effect of capitalist regimes of expropriation and accumulation on the societies they colonized are equally applicable to the behavior of the socialist Soviet state toward the entities it brought under its political control” (Kołodziejczyk and Şandru, 2012: 115): “[f]or “applied Marxism” (i.e., communism) was, like industrial capitalism, a purveyor of enforced modernization; many of its policies—rapid industrialization and urbanization, development of infrastructure, fight against religious prejudice, tribalism, and “traditional ways” (seen as barbaric by the colonial masters and “bourgeois” by the communists)—are similar to those deployed in newly-colonized countries” (Kołodziejczyk and Şandru, 2012: 115). Western intellectuals, who failed to recognize these similarities, often glorified the anti-capitalist, and thus allegedly anti-imperialist nature of the USSR. The latter was conveniently hiding Soviet mass murders and national cleansings^3^, political purges of national elites, ideological repressions, and denial of the right of colonized peoples to their national identification (labeled “bourgeois nationalism”) under the shallow surface of “the friendship of peoples.”

“The friendship of peoples”—or *druzhba narodov—*was a Soviet myth created and actively promoted by the USSR, which denied any prejudice within its borders based on national or ethnic background. The myth of “the friendship of (Soviet) peoples,” although it was a powerful tool of the Soviet propaganda machine—the same as the myth of the “Slavic Trinity” propagated now by the Russian Federation—proved to be a very inconsistent and unstable construction. The official Soviet foreign policy line condemned the inhuman working and living conditions as well as segregation laws targeted at African Americans, portraying Western racism as a constituent and integral part of the capitalist world (and, of course, not without a plethora of reasons). Ironically enough, while criticizing and denouncing American racism against the black population of the country, the Soviet Union (and later the Russian Federation) has persistently cultivated a rhetoric of despise toward ethnic non-Russians (predominantly dark-skinned and dark-haired migrants from the Caucasus and Central Asia who came to seek a better life in the big cities of the Russian Republic/Federation). In his study on racism in the late Soviet era, Jeff Sahadeo and his colleagues interviewed 70 migrants who were living in Moscow and St. Petersburg in the 1970s and 1980s and who shared their encounters with Russian racism, which encompassed the whole spectrum from “stares to epithets, from slights to violence” (Sahadeo, 2016: 803). It was (and still is) not uncommon for these people to be refused an apartment rental or to hear ugly insults that are predominantly based on their skin color: “blacks” (chernye), “black snouts” (chernomazye), or “black asses” (chernozlopy) (Sahadeo, 2016: 797), as well as “churki” (referring mostly to people of Asian origin). Thus, as Sahadeo points out, “[b]lackness became a discourse and a category with which to articulate the anxieties of European, or white, Russians toward growing migration from their former colonial peripheries” (Sahadeo, 2016: 797). These anxieties are rooted in the belief of Russian supremacy perpetuated by the Soviet Union, which framed Russians as “elder brothers” and nations inhabiting Central Asia and the Caucasus region as “backward little brothers,” thus making it unacceptable from the imperial perspective to get “(re)colonized” by the people who are regarded as inferior in Russian ethnic hierarchies.

### Terra nullius

Within the context of Russia’s colonialist paradigm, the Ukrainian language has consistently served as the quintessential marker of otherness for the Ukrainian people, highlighting national, cultural, and historical differences between the colonizers and the colonized. To erase these differences, Russian colonial policy systematically attempted to eliminate the Ukrainian language, which has survived over a hundred prohibitions and suppressions launched first by the Russian Empire and then by the Soviet Union. These prohibitions included the notorious Valuev Circular of 1863, which banned the publication of all popular literature, including textbooks and religious texts in Ukrainian. It was followed by the Ems Ukaz of 1876, which extended the prohibition to imports of any Ukrainian books into the territory of the Russian Empire from abroad, the creation of original works in Ukrainian, making translations from foreign languages into Ukrainian, and even the printing of text into musical notes. The colonial policy of Ukraine’s total Russification found its consistent and systematic continuation in the USSR. Although there was no official language in the Soviet Union, the Russian language was granted the status of intra-ethnic communication and acquired privileged status as the language of official media, science, university education, high-career positions, and everything associated with progress, modernity, and development. Ukrainian, on the other hand, just like other national languages, was artificially framed as the language of an uneducated, ignorant, and impoverished province, able to produce neither any works of high culture nor scientific knowledge^4^. This rhetoric was preserved in both Ukraine and the Russian Federation even after the collapse of the Soviet Union, with Russian media conveniently labeling Ukrainian-speaking people as barbarian “nationalists” or even “Nazis.” Moreover, the Kremlin has been defining anyone who, according to Vladimir Putin, “speaks and thinks in Russian” as a part of *russkii mir*, which proved to be a sufficient reason for the Russian government to launch the full-scale military invasion of Ukraine on 24 February 2022, with the aim to “demilitarize and denazify Ukraine.” Remarkably, in the temporarily occupied territories of Ukraine, the Russian Federation is deliberately pursuing its imperial policy of linguicide: Ukrainians are being persecuted based on their language, Ukrainian books are banned from libraries and schools, and Ukrainian toponyms are substituted with Russian ones.

While European colonizers have always *emphasized* an essentialistic dichotomy of “Occident-Orient,” Russian colonialism *denies* any differences between the colonizers and the colonized on the premise that the latter should surrender their identity and accept Russia as the universal norm. Ukrainian philosopher Volodymyr Yermolenko has defined a major difference between European and Russian colonial paradigms:

“Western-type colonialism was essential *racial*: it was built upon an idea that one group is “naturally destined” to be subordinated due to its race and skin color. It was an imperialism based on *fatal identity*. Eastern European colonialism differs: it was based on the “historical” idea that a people actually *can* – and should – change its place in the universe by renouncing their identity. In short, an imperialism of *changeable identity*.” (Yermolenko, 2021: 24; emphasis in the original).

Therefore, the colonized Ukrainian had to “renounce the less visible parts of their nation: language, literature, music, traditions, and so on” (Yermolenko, 2021: 24) to get rid of the stigma of being “inferior,” “barbarian,” and “second-class” in the social and political hierarchy of Russian colonialism, whose aggressive politics aimed at diminishing Ukrainian culture to the local stereotype of a “reduced model of the Orient, suitable for prevailing” (Said, 2019: 153) and consequently swallowing it together with its best samples and most prominent figures. That is exactly what happened to Volodymyr the Great—Prince of Kyiv and ruler of Kyivan Rus, Princess Anne of Kyiv, great Ukrainian artists Kazymyr Malevych and Illya Repin, and a lead rocket engineer in the USSR, Sergiy Korolev—to mention just a few names expropriated by Russian colonialism.

This desire first to disown Ukrainian history and culture from its Ukrainian origin and then to appropriate it has created a paradox in colonial logic when the colonizers actively claim their Ukrainian roots and celebrate separate pieces of Ukrainian culture, albeit in their rather simplified form. As was observed by Riabczuk, “Russians typically treat Ukrainians as a subgroup of their nation; they “love” Ukrainians as themselves, as an imperial myth, which is hardly acceptable for Ukrainians since it leaves no room for the latter’s separate national identity (not just a regional one, within the Greater Russian identity)” (Riabczuk, 2010: 8). Russian colonialism would even employ some Ukrainian national/aboriginal symbols and narratives to legitimize Russian imperial presence and influence in Ukraine, thus allowing some simplified forms of Ukrainian culture to be present in the official discourses. If the nation is an imagined community, as it was claimed by Benedict Anderson, the Ukrainian nation was re-imagined by Russian imperialism as an Orientalized, exotic “singing and dancing Little Russia” – an ambivalent geopolitical construct, in which “everything that is good […] comes from the common Rus/Russian legacy. Everything that is bad comes from evil, alien influences: Polish, Catholic, Jesuit, Uniate, or Tatar, Jewish, German, and so on” (Riabczuk, 2010: 14). Such a paradigm created an equivocal liminal space in the colonial discourse, which could simultaneously claim “brotherhood” with “Little Russians” and label Ukrainians as “extreme nationalists and neo-Nazis”^5^ that should be exterminated as soon as they voiced the desire to build their own nation-state outside of the imperialistic *russkii mir*. This colonial double standard has been skillfully summarized by Mykola Riabczuk:

“From a postcolonial point of view, Russian-Ukrainian relations may be compared to the relationship between Robinson Crusoe and Friday: every Robinson “loves” his Friday, but only as long as Friday follows the rules of the game established by Robinson, accepts colonial subordination, and does not question the superiority of Robinson and his culture.” (Riabczuk, 2010: 8–9).

Ukrainians’ refusal to accept their inferior role has undermined the established order of the Manichean world created by Russian colonialism, thus posing a direct threat to Russian dominance in the region. The reaction to this disobedience was the complete negation of Ukraine’s statehood and its agency, which was manifested in Putin’s notorious speech on 21 February 2022: “[M]odern Ukraine was entirely created by Russia or, to be more precise, by Bolshevik, Communist Russia^6^.” The refusal of Ukraine’s statehood and nationhood here serves two purposes. First, if Ukraine “was entirely created by Russia,” the Russian military occupation of sovereign Ukraine would only amend an unfortunate historical misunderstanding that resulted in the latter’s independence. Second, by refusing Ukraine’s statehood, Putin sets a logic according to which the occupation of Ukraine is not really an occupation since there is no state to occupy—just a piece of land, *terra nullius*, populated by people without national identity—the same rhetoric was employed by Hitler before and during the Nazi occupation of Poland.^7^

### The orientalized Ukraine

The long repudiation of Ukraine’s statehood by Russian imperialism was, consciously or not, accommodated by Western societies. The annexation of Ukraine’s Crimea and the start of the war in the Donetsk and Luhansk oblasts, both of which were initiated by the Russian Federation in 2014 as the first stage of the Russian neo-colonial project, were regarded as a domestic conflict rather than a direct and open threat to global democracy and peace. The war in Ukraine and its further escalation in February 2022 shattered the long-established *status quo* that conveniently defined Ukraine as “an in-between zone ‘East of Europe, West of Russia’” (Mälksoo, 2022: 3). The fear of a possible direct confrontation with the aggressor state and the nuclear anxiety engendered by Putin’s threats of nuclear escalation stirred up calls for peace and denials to send military aid and weapons to Ukraine. Together with paranoid “[i]nsinuations of the dangers of Ukrainian ‘nationalism’”^8^ (Mälksoo, 2022: 2), this pacifistic stance effectively negated the political rights of the Ukrainian nation, thus following a pattern of rhetoric set by Russian propaganda that has been consistently framing Ukraine as a “NATO puppet state” fully stripped of its own agency and ruled by foreign forces. An example of such a framing is the very first sentence of Santoire’s (2022), article “A Feminist Reality Check on Ukraine,” published on 14 February 2022, in which the author claims that “Ukraine has become a dangerous playground for great powers as Western countries and NATO are increasingly worried about renewed hostilities in the Donbas war.” Such a definition completely denies Ukraine’s agency as a sovereign state, reducing it to a passive, inert “playground” and rejecting the centuries-long history of Russian colonial aggression against Ukraine. Moreover, naming “the Donbas War” diminishes the war waged on Ukraine by the Russian Federation in 2014 as a local “conflict of interest,” thus turning a blind eye to Kremlin’s direct responsibility for the violation of international laws and Ukraine’s territorial integrity.

The countries of Central Eastern Europe that were part of the Soviet bloc, in general, have been traditionally looked down upon by the West as a “contemporary periphery”: ‘“European enough’ (geographically), ‘yet not advanced enough’ to become ‘Western’ (temporarily)” (Kulpa and Mizielinska, 2011: 18). If “the ‘hegemonic temporality of West’ is constructed as continuous and linear, progressive and ‘accumulative’” (Kulpa and Mizielinska, 2011: 18), Eastern Europe after 1989 found itself in a “knotted time” (Kulpa and Mizielinska, 2011: 16). In this paradigm, Ukraine has been regarded as a periphery of a periphery—even less civilized, less cultured, less developed, and more dependent on Russian policy than the other countries of the region. Russian colonialism has for centuries represented Ukraine as its exotic, semi-wild, archaic southern borderland. The collective West has easily adopted the same perspective of Ukraine and Ukrainian people as a *fait accompli* without even attempting to question its logic and validity. Such ignorance is a prime example of the legacy of the European colonial past and (neo)colonial thinking that has survived till our days: colonial powers recognize only other colonial powers, ignoring the voices of those colonized and rejecting their agency. This attitude could be found in the predictions that Kyiv would fall within 96 h after the start of the invasion, in hesitations of military supply delivery to Ukraine during the first days of the invasion and in numerous appeals to stop supplying Ukraine weapons. During the first weeks of the full-scale invasion, feminists from Croatia^9^ and Germany^10^ published open letters, which, together with the international manifesto “Feminists Against War,” were urging their governments to stop supporting Ukraine with weapon supplies, thus denying Ukrainians one of the very basic human rights, the right to resist and protect their lives and their country. All three appeals mentioned are not only made from the position of Western privilege; they are of a deeply colonial nature since the authors of the writings have never consulted Ukrainian feminists (and later ignored the just criticism from the Ukrainian feminist community), thus claiming themselves the status of “a white savior” who always knows better. In response to these claims, Ukrainian feminists have released “The right to resist”^11^ manifesto that condemns “abstract pacifism” that does not differentiate between Russia as the perpetrator state and Ukraine as the state that has to protect its own sovereignty. This manifesto resonates with the statement of the Kurdish feminist Dirik (2017), who, in her article “Feminist pacifism or passive-ism?” also criticized Western feminists’ impotence to “qualitatively distinguish between statist, colonialist, imperialist, interventionist militarism and necessary, legitimate self-defence.” Frantz Fanon, a pioneering postcolonial theorist and activist, also recognized the same right of the colonized to defend themselves against the colonizer, who defined decolonization as “always a violent phenomenon” (Fanon, 2001: 27).

Western feminists often explain their reluctance to support the demands of Ukrainian feminists in favor of the weapons supply to the country with the rhetoric that military support of Ukraine will reinforce anti-gender and patriarchal discourses while reducing the role of women in society. Such claims are also a result of seeing Ukraine as an Orientalized space, which is *always already* pre-defined by Western knowledge (or, in this case, by the lack of such knowledge) and thus denies the possibility of development and transformation. Chandra Mohanty, in her essay “Under Western Eyes,” had at length criticized the production of the Third World woman as a “singular monolithic subject” (Mohanty, 1988: 61) in the Western feminist discourses, which often reduced the variety of complexity of these women’s experiences to a convenient image of “an average third world woman”: (“ignorant, poor, uneducated, tradition-bound, religious, domesticated, family-oriented, victimized, etc.)” (Mohanty, 1988: 65). Mohanty warns that such an “application of the notion of women as a homogeneous category to women in the third world colonizes and appropriates the pluralities of the simultaneous location of different groups of women in social class and ethnic frameworks; in doing so, it ultimately robs them of their historical and political *agency*” (Mohanty, 1988: 79, emphasis in the original). Although Mohanty talks about Third World women’s experiences, the same is true about Ukrainian women. Moreover, if women of the Third World could at least locate themselves in discursive debates, women of the former Soviet bloc countries have found themselves in a theoretical void engendered after the collapse of the Soviet Union, which surrounded the Russian Federation as the ultimate subject of academic interest for many Western intellectuals. Although it remains difficult for many postcolonial theorists to recognize the postcolonial dynamic between the Russian Federation as the (former) metropolis and the nation-states colonized by it^12^, producing and re-presenting a composite singular image of Ukrainian womanhood by the Western feminist discourses mirrors the same patterns in relations of power condemned by Mohanty.

### Ukrainian women under Western eyes

However, there was a significant difference in Western production of Ukrainian women^13^ that distinguishes her from Third World women—her liminality. The Iron Curtain that separated the Soviet Union from the rest of the world has also become an Orientalist veil for Ukrainian women, and it did not completely disappear after the fall of the Soviet empire. Coming from a place that was known as the Second World War, she finds herself located in a liminal time and space—somewhere between Eastern Europe and another province of the Russian Federation, forever stuck in the post-Soviet temporality. Such a positionality creates a logical paradox that defines Ukrainian women as static, monolithic objects, which, at the same time, can be assigned a fluid identity: they are simultaneously seen as passive victims of the war and as objects of racialized fear, as exotic, and as those who are privileged because of their skin color. The fact that Ukrainian refugees receive treatment in the EU that is different from that of non-white refugees coming from Syria, Iraq, Afghanistan, and other countries is undeniable and exposes the existing system of racism in Europe. However, labeling Ukrainians as “privileged refugees”^14^ not only denies their experiences as victims of the terrible war of extinction but also ignores the centuries-long history of occupation and exploitation of Ukraine by Russian colonialism. It is important to realize that “this whiteness granted to Ukrainians by Western European and North American narratives is temporal and conditional” (Lambert, 2023: 20) and can be a little controlled by Ukrainians themselves.

Ukraine is not a homogeneously white country as it is imagined to be by Western societies that often ignore the heterogeneity of Ukrainian women, who can also be of Jewish, Roma, or Crimean Tatar origin, thus reducing them to a compact stereotypical image of a Ukrainian as a “blue-eyed” savage. I want to illustrate my argument with a prominent (although exaggerated) example from popular culture: a Ukrainian woman named Petra, who was portrayed in one of the episodes of a popular Netflix show, *Emily in Paris*.^15^ Petra is an example of the Orientalized Ukrainian woman who has entered the civilized space of one of the greatest European imperial capitals, Paris. Petra is a stereotypical “blond and blue-eyed” Ukrainian woman who cannot speak any other language except Ukrainian, steals goods from the local shop, and is ready to fight with Emily, the protagonist when she insists on returning the stolen and is anxious about being deported from France. Petra is a hyperbolic caricature of a Ukrainian savage that has entered civilized European space and is menacing to destroy it if not controlled by Emily, who functions as an agent of Western (specifically American) values and norms. Petra’s personality is diminished in two aspects: she is Ukrainian and enjoys shoplifting. The fact that Petra is not even a Ukrainian name only reconfirms my claim that the West has (re)created a distorted image of Ukrainian women based on the double colonial perspective and the lack of knowledge it has engendered.

Before the dissolution of the USSR, Ukrainian women did not exist in the imagination of Westerners. Instead, there was a monolithic figure of the Soviet Superwoman, who was exempt from any cultural or national distinctions and expected to fit in with the colonial Iron Maiden. A Ukrainian scholar, Hrytsenko (2022), observes that “the differences between women’s history and culture, women’s experiences, and women’s needs, more elaborated than “Muscovites have two pigtails, Uzbeks have twenty-five,” were erased and adjusted to an averaged specimen standing much closer to Muscovites than Uzbeks,” thus making a Muscovite a universal archetype of a Soviet woman. After 1991, Ukraine appeared on the international geopolitical stage as an independent country, and Ukrainian women could finally be unveiled as subjects of the sovereign state. The Soviet Superwoman model was challenged by the new values, norms, and lifestyles that flooded the country after the fall of the Iron Curtain. These new tendencies and influences have re-fashioned the ideals of female identity and produced two new models of Ukrainian femininity, which Ukrainian scholar Oksana Kis has defined as *Berehynia* and *Barbie* (Kis, 2005). Berehynia (a woman-guardian) is a female archetype whose main functions and roles were associated with a woman’s reproduction, nurturing, and mothering abilities, whereas Barbie is an embodiment of female sex appeal fully situated on the surface of a woman’s body, implying that a woman’s main purpose is to be an object of male desire capable of satisfying male gaze. In the 1990s, Berehynia and Barbie were mostly confined to the domestic space, but in the 2000s and early 2010s, they entered the public sphere of Ukraine’s social life. As pointed out by Rubchak (2005) and Кісь (2012), the female voice of the Orange Revolution of 2004 was Yuliia Tymoshenko, an oppositional Ukrainian politician who actively utilized the Berehynia image in her political campaigns.

However, Barbie has also stepped out of the domesticity realm and employed her sexuality to challenge and deconstruct the patriarchal image of Ukrainian womanhood. The best example of such opposition is the radical feminist activist group *Femen,* notorious for their provocative topless protests and ideology of “sextremism, atheism, and feminism” (Reestorff, 2018: 207). The Revolution of Dignity of 2014 has only reinforced the tendency for women’s emancipation and integration into the public sphere.

Although Berehynia and Barbie archetypes were still strongly present on the Revolution’s political stage, women were primarily expected either to perform nurturing and maternal roles or to inspire male protesters with their beauty and youth. The Revolution of Dignity has produced a new kind of Ukrainian femininity—an active woman-agent who managed to find her place in the male militarism of the protests (Martsenyuk, 2014; Phillips, 2014). Thus, the Revolution of Dignity has sparked significant transformations in Ukrainian femininity. This fight for European democratic values has marked a new era of society’s development, which is now characterized by the processes of what Ukrainian philosopher Tamara Zlobina has defined as *gender decay*^16^ —the rejection of old gender models and the conception of a new emancipatory social rhetoric, which has allowed high social mobility and visibility of women in spheres traditionally associated with men: politics, military services, business, and volunteering. Symbolically enough, breaking into the colonial past of Russian rule and choosing a pro-European path of development also meant for Ukrainian society to break with the established patriarchal norms and gender roles.

However, these profound and complex changes in the Ukrainian social and political landscape remained mainly unnoticed by Western societies, which mainly imagined Ukrainian women as a combination of Berehynia and Barbie archetypes that were Orientalized into *Housemaid* and *Jezebel.* While Jezebel is a stereotypically beautiful and sexually appealing woman desperate to capitalize on her beauty in the world of Western capitalism, Housemaid (when her sexuality is tamed and controlled) can be a good material for performing care labor due to her “innate” submissiveness and lack of professional ambition. Such a paradigm was reinforced in the Western imagination by two facts: first, most Ukrainian women working in the European Union usually provided reproductive work services (e.g., childcare, elderly care, and housekeeping), and second, many women involved in the sex industry in the European Union were coming from the East-European region^17^—a region that was stripped of national differences and mostly regarded as post-Soviet—and thus belonging to the Russian area of influence.

### Anticolonial struggle

The Russian full-scale war against Ukraine catalyzed the emancipatory processes even more. Ukrainian women have shown their readiness to defend their country in this neo-colonial struggle for existence. There are more than 60,000 women serving in the Ukrainian Armed Forces, of which approximately 5,000 are currently on the front lines of the war. Together with millions of women volunteers, medical professionals, journalists, activists, educators, etc., they have resisted Russian neo-colonialism as active agents, refusing to be classified as a homogeneous group of powerless victims of colonial violence. Moreover, I argue that the active participation of Ukrainian women in defense of the Ukrainian state and their further emancipation is a part of the anticolonial struggle against the ideology of r*usskii mir,* which is heavily loaded with and largely dominated by discourses of hetero-patriarchy and heteropaternalism. The very symbol of this power hierarchy is Vladimir Putin himself, whose bare-chested image of riding a horse somewhere in Siberia embodies the hegemonic project of hypermasculinity and male dominance established in Russian society. Elisabeth Wood points out that “[Putin’s] persona stands out as gendered in three distinct registers: visual imagery (the Russian Marlboro Man); domination of the political sphere through verbal attacks on other men; and a series of crude, macho aphorisms that have been collected as ‘Putinisms’” (Wood, 2016: 2). The fascist regime currently dominating Russian society is deeply rooted in Putin’s persona, which has engendered a cult of machoism as the source of political power.

While the Russian Federation has officially decriminalized domestic violence (February 2017) and passed the law that bans “LGBT propaganda” among adults (November 2022; this followed the 2013 “Gay Propaganda” law that prohibited the dissemination of information about “non-traditional” sexual relationships to minors and was misused to suppress LGBTQ+ rights and activism), the Parliament of Ukraine has ratified the Istanbul Convention and unanimously passed a bill banning hate speech against LGBTQ+ people in the media. These two changes to Ukraine’s legislation were adopted after the Russian full-scale invasion of Ukraine (in July and December, respectively), thus also denoting a symbolic separation from the values system of *russkii mir.* Ukrainian women and LGBTQ+ people who are fighting against Russian invaders undermine the very foundation of the colonial worldview, which allows and recognizes only rigid gender roles established by hegemonic heteronormativity and hypermasculinity.

### Instead of conclusion

Having been seen through a double colonial lens—the long-standing tradition of Russian colonialism and Western Orientalism—Ukraine and Ukrainians have been (re)produced in a crooked mirror, none of the images reflecting what they truly are. Russian colonialism has invented the image of exotic Little Russians—subhuman “brothers” of Great Russians; the West has seen Ukrainians as underdeveloped barbarians somewhere between Russia and European civilization. The resistance of Ukrainians since 24 February 2022 has been challenging both of these images. Since February, Ukrainian society is going through an accelerated decolonizing process, which encompasses not only social, cultural, and political spheres but has also penetrated gender and identity forming discourses. Although this is only the beginning of a long process, I believe we are on the right path.

## Author contributions

The author confirms being the sole contributor of this work and has approved it for publication.

## Footnotes

^1^For example, Frantz Fanon in his *The wretched of the earth* adapts Marxist vocabulary and regards imperialism as a project of “colonial bourgeoisie” (Fanon, 2001: 38).

^2^I am referring here to the title of Vladimir Lenin’s work *Imperialism, the highest stage of capitalism: a popular outline* (1917).

^3^Just to mention a few examples: the Polish Operation of 1937–1938, the mass deportation and killing of Crimean Tatars in May 1944, or Holodomor – a man-made Terror-Famine of 1932–1933, which targeted ethnic Ukrainians and Kazakhs and took millions of people’s lives.

^4^Such aggressive language policies are common for colonial occupation: e.g., the brutality the Irish language has experienced under the British colonial violence or the repressions of the Basque language during the Francoist dictatorship.

^5^Vladimir Putin’s speech as of February 24, 2022, before the start of the invasion: https://www.spectator.co.uk/article/full-text-putin-s-declaration-of-war-on-ukraine/

^6^Full transcript of the speech: https://mandynews.com/putin-speech-on-ukraine/

^7^Timothy Snyder extensively engages with the topic of the destruction of Poland’s statehood by Nazi Germany in his book “Black Earth: The Holocaust as History and Warning” (2015).

^8^To compare: the far right’s share in the Parliament of Ukraine according to the results of the parliamentary elections of 2019 is 2% (source: https://www.factcheck.org/2022/03/the-facts-on-de-nazifying-ukraine/), while Italy’s far right leader Giorgia Meloni has gained 26% of support during the 2022 elections (https://www.bbc.com/news/world-europe-63029909), France’s nationalist and far right Le Pen has got 41.46% competing with Emmanuel Macron during the presidential elections of 2022 (https://www.cnbc.com/2022/04/25/macron-wins-election-but-frances-far-right-has-gotten-record-support.html), and German far right party AfD has won 10.3% of share in the German parliament during 2021 elections (https://www.theguardian.com/world/ng-interactive/2021/sep/26/german-election-results-exit-poll-and-possible-coalitions).

^9^Ukraine: Women’s Appeal for Peace: https://www.europe-solidaire.org/spip.php?article61400

^10^Open Letter to Olaf Scholz: https://www.emma.de/artikel/open-letter-chancellor-olaf-scholz-339499

^11^Right to Resist manifesto: https://commons.com.ua/en/right-resist-feminist-manifesto/

^12^This includes not only nations that established independence after the collapse of the Soviet Union, but also nations that remain occupied by the colonial power: Chechens, Dagestani, Komi, Sakha, Chuvash and many others.

^13^See: Chandra Mohanty’s thoughts on “Woman” vs. “women” in her article “Under Western Eyes.”

^14^https://www.dhakatribune.com/opinion/op-ed/299568/are-there-any-privileged-refugees

^15^*Emily in Paris*: season 2, episode 4.

^16^Lecture “Gendernyi rozpad.” (2016). Available at https://www.youtube.com/watch?v=RN7-9PVzJYc

^17^Report “Sex Work in Europe,” 2009: https://tampep.eu/wp-content/uploads/2017/11/TAMPEP-2009-European-Mapping-Report.pdf

## References

[ref1002] Ageyeva V. (2021). Behind the scenes of the empire. Kyiv: Vikhola.

[ref1] Ashcroft B. Griffiths G. Tiffin H. (2003). The postcolonial studies reader. London: Routlage.

[ref2] Dirik D. (2017). Feminist pacifism or passive-ism? *Open Democracy*. Available at: https://www.opendemocracy.net/en/5050/feminist-pacifism-or-passive-ism/?fbclid=IwAR1ztKa1cTFMz4cN_K-KI330HrluDQZ2wnRVoeLe7jFZkEBRu3VUwl8SMK8

[ref3] Fanon F. (2001) The wretched of the earth. London: Penguin Books.

[ref4] Hrytsenko H. (2022). Are we all sisters? Ukrainian feminism between the west and Russia*. Gender in detail*. Available at: https://genderindetail.org.ua/season-topic/dosvidy-viyny/are-we-all-sisters-eng.html

[ref14] Кісь O. (2012). Кого оберігає берегиня, або матріархат як чоловічий винахід. *Gender in Detail*. Available at: https://genderindetail.org.ua/library/ukraina/kogo-oberigae-bereginya-abo-matriarhat-yak-cholovichiy-vinahid-1341287.html (Accessed March 21, 2022).

[ref5] Kis O. (2005) in Choosing without choice: dominant models of femininity in contemporary Ukraine. *Gender transitions in Russia and Eastern Europe*. eds. Hurd M. Carlback H. Rastback S. (Stockholm: Gondolin Publishers), 105–136.

[ref6] Kołodziejczyk D. Şandru C. (2012). Introduction: on colonialism, communism and east-Central Europe – some reflections. J. Postcol. Writ. 48, 113–116. doi: 10.1080/17449855.2012.658242

[ref7] Kulpa R. Mizielinska J. (2011). “‘Contemporary peripheries’ – queer studies, circulation of knowledge and east/west divide” in De-Centring Western sexualities: central and eastern European perspectives. eds. Kulpa R. Mizielinska J. (London: Routledge), 11–27.

[ref8] Lambert L. (2023). Fifty shades of white(ness): introduction. Funambulist 48, 20–23.

[ref9] Mälksoo M. (2022). The postcolonial moment in Russia’s war against Ukraine. J. Genoc. Res. 1–11. doi: 10.1080/14623528.2022.2074947

[ref10] Martsenyuk T. (2014). Гендерна соціологія Майдану: роль жінок в протестах. *Постсоціалістичні суспільства: різноманіття соціальних змін: матеріали Міжнар. соціологічних читань пам’яті Н.В. Паніної та Т.І. Заславської*, Available at: http://ekmair.ukma.edu.ua/handle/123456789/3511.

[ref12] Mohanty C. (1988). Under Western eyes: feminist scholarship and colonial discourses. Fem. Rev. 30, 61–88. doi: 10.1057/fr.1988.42

[ref13] Moore D. (2001). Is the post- in postcolonial the post- in post-soviet? Toward a global postcolonial critique. PMLA 116, 111–128. doi: 10.1632/pmla.2001.116.1.111

[ref15] Pease E. D. (2005). “US imperialism: global dominance without colonies” in A companion to postcolonial studies. eds. Schwarz H. Ray S. (Oxford: Blackwell Publishing), 203–220.

[ref16] Phillips S. D. (2014). The women's squad in Ukraine's protests: feminism, nationalism, and militarism on the Maidan. Am. Ethnol. 41, 414–426. doi: 10.1111/amet.12093

[ref25] Riabczuk M. (2010). The Ukrainian “Friday” and the Russian “Robinson”: the uneasy advent of Postcoloniality. Canadian–American Slavic studies. 7–24. Available at: https://brill.com/view/journals/css/44/1-2/article-p7_2.xml

[ref18] Riabczuk M. (2013). Colonialism in another way. On the applicability of postcolonial methodology for the study of postcolonial Europe. Porównania 13, 47–59. doi: 10.14746/p.2013.13.10972

[ref19] Rubchak M. J. (2005). Yulia Tymoshenko: goddess of the Orange revolution. *Transitions Online*. Available at: http://www.tol.org/client/article/13378-goddess-of-the-orange-revolution.html.

[ref20] Reestorff C. M. (2018). “Affective activism and political secularism: the unending body in the Femen movement” in Routledge companion to media and activism. ed. Meikle G. (Abington: Routledge)

[ref21] Said E. (1994). Culture and Imperialism. London: Vintage.

[ref22] Said E (2019). Orientalism. London: Penguin.

[ref23] Sahadeo J. (2016). Black snouts go home! Migration and race in late soviet Leningrad and Moscow. J. Mod. Hist. 88, 797–826.

[ref24] Santoire B. (2022). Feminist reality check on the Ukraine crisis. *The Research Network on Women, Peace and Security*. Available at: https://www.mcgill.ca/rnwps/article/our-blog/feminist-reality-check-ukraine-crisis

[ref26] Velychko S. (2002). The issue of Russian colonialism in Ukrainian thought. Dependency identity and development. Ab Imperio 1, 323–367. doi: 10.1353/imp.2002.0070

[ref28] Wood E. (2016). Hypermasculinity as a scenario of power. Int. Fem. J. Polit. 18,3. doi: 10.1080/14616742.2015.1125649

[ref29] Yermolenko V. (2021). Atypical post-colonialism: Ukraine in global political thought. UA 2, 19–25.

